# Novel Expressed Sequence Tag-Derived and Other Genomic Simple Sequence Repeat Markers Revealed Genetic Diversity in Ethiopian Finger Millet Landrace Populations and Cultivars

**DOI:** 10.3389/fpls.2021.735610

**Published:** 2021-09-23

**Authors:** Haftom Brhane, Teklehaimanot Haileselassie, Kassahun Tesfaye, Cecilia Hammenhag, Rodomiro Ortiz, Kibrom B. Abreha, Mulatu Geleta

**Affiliations:** ^1^Institute of Biotechnology, Addis Ababa University, Addis Ababa, Ethiopia; ^2^Ethiopian Biotechnology Institute, Ministry of Science and Technology, Addis Ababa, Ethiopia; ^3^Department of Plant Breeding, Swedish University of Agricultural Sciences, Lomma, Sweden

**Keywords:** amphidiploid, expressed sequence tag, finger millet, genetic diversity, molecular markers

## Abstract

Finger millet (*Eleusine coracana* (L.) Geartn.) is a self-pollinating amphidiploid crop cultivated with minimal input for food and feed, as well as a source of income for small-scale farmers. To efficiently assess its genetic diversity for conservation and use in breeding programs, polymorphic DNA markers that represent its complex tetraploid genome have to be developed and used. In this study, 13 new expressed sequence tag-derived simple sequence repeat (EST-SSR) markers were developed based on publicly available finger millet ESTs. Using 10 polymorphic SSR markers (3 genomic and 7 novel EST-derived), the genetic diversity of 55 landrace accessions and 5 cultivars of finger millet representing its major growing areas in Ethiopia was assessed. In total, 26 alleles were detected across the 10 loci, and the average observed number of alleles per locus was 5.6. The polymorphic information content (PIC) of the loci ranged from 0.045 (*Elco-48*) to 0.71 (*UGEP-66*). The level of genetic diversity did not differ much between the accessions with the mean gene diversity estimates ranging only from 0.44 (accession 216054) to 0.68 (accession 237443). Similarly, a narrow range of variation was recorded at the level of regional states ranging from 0.54 (Oromia) to 0.59 (Amhara and Tigray). Interestingly, the average gene diversity of the landrace accessions (0.57) was similar to that of the cultivars (0.58). The analysis of molecular variance (AMOVA) revealed significant genetic variation both within and among accessions. The variation among the accessions accounted for 18.8% of the total variation (*F*_*ST*_ = 0.19; *P* < 0.001). Similarly, significant genetic variation was obtained among the geographic regions, accounting for 6.9% of the total variation (*P* < 0.001). The results of the cluster, principal coordinate, and population structure analyses suggest a poor correlation between the genetic makeups of finger millet landrace populations and their geographic regions of origin, which in turn suggests strong gene flow between populations within and across geographic regions. This study contributed novel EST-SSR markers for their various applications, and those that were monomorphic should be tested in more diverse finger millet genetic resources.

## Introduction

Finger millet (*Eleusine coracana* G.), which is commonly called “Ragi” in India and “Dagusa” in Ethiopia, belongs to the subfamily Chloridoideae in the family Poaceae. It is a self-pollinated (95%) amphidiploid (2*n* = 4*x* = 36; with AABB genome) cereal crop cultivated in tropical and subtropical parts of Africa and Asia (Dida et al., 2008; Goron and Raizada, 2015). A flow cytometric analysis has estimated the genome size of finger millet (1C value) to be about 1.9 pg (Mysore and Baird, 1997), which is approximately 1.86 Gbp according to the conversion factor provided in the study by Dolezel et al. (2003). The assembly of 1.18 Gbp (Hittalmani et al., 2017) of the finger millet genome is available at https://www.ncbi.nlm.nih.gov/assembly/GCA_002180455.1/. Some archeological records suggested that the cultivation of finger millet started in Ethiopia and Uganda, and later reached India during the second millennium BC (Hilu and Dewet, 1976). Finger millet is the third most important cereal crop in semiarid areas of the world, only surpassed by sorghum and pearl millet (Barbeau and Hilu, 1993). It is cultivated as a food crop for its nutritious grain with additional use of its straw as livestock feed. It is an important source of calcium, iron, essential amino acids, and dietary fiber, as well as a health-promoting substance such as anti-hypocholesterolemia, anti-hypoglycemia, and anti-ulcer compounds (Barbeau and Hilu, 1993; Chethan and Malleshi, 2007; Nakarani et al., 2021). It also serves as a source of additional income for smallholder farmers. Finger millet is recognized as a promising climate-resilient crop combining the ability to perform well on marginal lands and under moisture, salt, and acidity stress conditions and good storage quality and high nutritional value (Dida et al., 2007). However, it is one of the few research-neglected crops globally and consequently referred to as an “orphan” crop.

In Ethiopia, it is the sixth most important cereal crop after teff, wheat, maize, sorghum, and barley (Fentie et al., 2013). Its average grain yield in Ethiopia is about 2 tha^−1^, which can be regarded as low, but it has the potential to yield up to 3 tha^−1^ (CSA, 2016). Factors such as limitedly improved cultivars, not adopting new technologies, lack of agronomic packages, lodging, drought, and diseases such as head blast are major contributors to its low yield (Degu et al., 2009; Molla, 2010). Hence, overcoming these constraints including the development of new improved cultivars tolerant to biotic and abiotic stresses can boost the grain yield of the crop for its significant contribution to food security.

Finger millet genomic resources and tools including its whole genome sequence published in 2017 by Hittalmani et al. (2017) are highly important for efficient genetic improvement and conservation of the crop. However, a significant proportion of published research outputs in finger millet have limitations in terms of sample size, representativeness of diverse ecological conditions or type, and the number of markers used. In the case of Ethiopian finger millet germplasm, a few research publications used morphological (Tsehaye and Kebebew, 2002; Daba and Debelo, 2008; Tesfaye and Mengistu, 2017; Kebede et al., 2019) and DNA markers such as random amplified polymorphic DNA (RAPD) (Babu et al., 2007) and inter simple sequence repeats (ISSRs) (Brhane et al., 2017). Hence, further genetic diversity assessment is needed for crucial insight into the gene pool of the crop in the country. Simple sequence repeat (SSR) markers are among the most preferred molecular markers for population genetics analyses and have been widely used in various crops such as finger millet (Babu et al., 2014; Manyasa et al., 2015; Ramakrishnan et al., 2016; Lule et al., 2018; Pandian et al., 2018; Prabhu et al., 2018). They have been useful for molecular breeders and geneticists to associate the phenotype-genotype variations for marker-assisted selection of desired genotypes (Babu et al., 2018).

Among the SSR markers, the expressed sequence tag-derived simple sequence repeats (EST-SSRs) have become popular due to their various advantages such as high transferability among closely related taxa, relative ease, and cost-effectiveness to develop (Teshome et al., 2015; Chombe et al., 2017; Gadissa et al., 2018; Serbessa et al., 2021). Due to the fact that they represent expressed parts of a genome, they have a higher average rate of transferability across species than genomic SSRs (Gupta et al., 2003), and they are highly associated with differentially expressed genes (Saha et al., 2004). The identification of candidate genes as an input for breeding and conservation, and the analyses on population genetics are among the various applications of EST-SSR markers (Yu et al., 2011). In this study, new EST-SSR markers were developed from publicly available finger millet EST sequences as a contribution to the genetic improvement of the crop. Using these new markers and previously developed genomic SSR markers (Dida et al., 2007), the genetic diversity and the population structure of finger millet representing four geographical regions in Ethiopia were assessed to determine the extent and distribution of its genetic diversity in the country.

## Materials and Methods

### Plant Material and DNA Extraction

Sixty finger millet accessions were used in this study. A total of 55 accessions were landraces originally collected from four regional states of Ethiopia (i.e., 15 from Amhara, 15 from Oromia, 15 from Tigray, and 10 from Southern Nations, Nationalities, and People's region) whereas the remaining five were improved cultivars. The landrace accessions and cultivars were obtained from the Ethiopian Biodiversity Institute (EBI) and the Bako Agricultural Research Center (BARC), respectively (Supplementary Table 1). Seeds of each accession were planted in a greenhouse at the Swedish University of Agricultural Sciences (SLU), Alnarp, Sweden. Leaf tissue was collected from young seedlings at about 3 weeks after planting, for DNA extraction. Each accession was represented by 15 individual plants. However, the samples of 29 accessions were collected individually (i.e., each accession was represented by 15 separate samples) whereas the samples of 31 accessions were collected in the pool (i.e., each accession was represented by a single pool of leaf tissue from 15 plants). Hereafter, the two groups of samples will be referred to as “indiv-accessions” and “pool-accessions,” respectively, for the sake of simplicity. For sampling, we used a deep-well plate containing two glass beads in each well. Immediately after sampling, the plate was sealed with a perforated lid, placed in a freeze drier for 4 days, and stored at −80°C until DNA extraction. Frozen samples were homogenized using a Retsch MM400 shaker (Haan, Germany) at 300 Hz for 30 s. DNA was extracted from the homogenized tissue using QIAcube HT (QIAGEN GmbH, Hilden, Germany) following a modified CTAB (Cetyltrimethylammonium bromide) procedure as described previously (Bekele et al., 2007; Tesfaye et al., 2007). A NanoDrop ND-1,000 spectrophotometer (Saveen Werner, Sweden) was used for estimating the quality and quantity of the DNA.

### The Development of New EST-SSR Markers

A total of 1,956 finger millet EST sequences downloaded from the National Center for Biotechnology Information (NCBI) were analyzed for the presence of SSRs within the EST sequences using a web-based software WebSat (Martins et al., 2009). A total of 100 sequences that contained SSRs with 2–6 repeat motifs were identified among the downloaded ESTs. After excluding duplicates, overlapping and very short sequences, and sequences with more than one SSRs, 50 EST sequences suitable for designing primers were maintained. Primer pairs were designed for the 50 ESTs targeting the SSRs using a web-based Primer3 primer-designing program version 4.1.0 (Rozen and Skaletsky, 2000). Of note, 10 samples from diverse finger millet accessions were used to evaluate the primer pairs for the quality of their amplified products. This led to the selection of 13 primer pairs that specifically amplified their target SSR loci. These primer pairs were used to amplify the target EST-SSRs (for PCR conditions, see the “SSR-PCR amplification and capillary electrophoresis” section). The amplified products of the 13 loci (five samples each) were purified using the GeneJet PCR Purification Kit (Thermo Fisher Scientific, Vilnius, Lithuania) followed by the Sanger sequencing to confirm that their sequence matches the original EST sequences. The sequencing was performed at Eurofins (www.eurofins.com) using a mixture of 2 μl of 10 μM of a sequencing primer and 15 μl of a purified PCR product. Each amplified product was sequenced using both forward and reverse primers. The comparative analysis of the EST sequences containing these target EST-SSRs through the Basic Local Alignment Search Tool (BLAST) was also performed to evaluate the transferability of the repeat motif across the Poaceae species and to determine the position of the SSRs within the corresponding genes.

### SSR-PCR Amplification and Capillary Electrophoresis

A total of 10 genomic SSRs reported to be highly polymorphic in the previous research (Dida et al., 2008) were screened for good amplification, polymorphism, specificity, and suitability for multiplexing using the same set of DNA samples used for testing the EST-SSRs, and three SSR loci were selected. Hence, the 13 EST-SSRs developed in this study and the 3 genomic SSRs from the study by Dida et al. (2008) were used to analyze the 60 finger millet accessions. The PCRs were performed in a volume of 25 μl containing 25 ng genomic DNA, 0.3 μM of each primer, 2 mM MgCl_2_, 0.3 mM dNTPs, 1 U Taq polymerase, and 1 × PCR buffer (10 mM Tris–HCl, pH 8.3, and 50 mM KCl). S1000™ Thermal Cycler (Bio-Rad, Hercules, CA, USA) was used for the amplification of the target loci using the following temperature profile: initial denaturation at 95°C for 5 min followed by 35 cycles of 30-s denaturation at 95°C, 30-s primer annealing at optimized temperature for each primer pair (ranging from 55°C to 59°C), and 1 min primer extension at 72°C, with a final primer extension at 72°C for 10 min.

The forward primers were 5′-labeled with 6-FAM™ or HEX™ fluorescent dyes. To prevent the non-template addition by Taq polymerase to the PCR products, the reverse primers were PIG-tailed with *GCTTCT* according to the study by Ballard et al. (2002). Prior to capillary electrophoresis, PCR amplification was confirmed by running 5 μl of the PCR products on 1.5% agarose gel containing GelRed and visualized using BioDoc-It™ Imaging System (Upland, CA, USA). The multiplexing of the PCR products was performed as described in the study by Geleta et al. (2012). The capillary electrophoresis of the PCR products was performed using an Applied Biosystems 3,500 Genetic Analyzer (Thermo Fisher Scientific, Waltham, MA, USA) at the Department of Plant Breeding, Swedish University of Agricultural Sciences, Sweden.

### Data Analysis

After capillary electrophoresis, GeneMarker version 2.7.0 (SoftGenetics, LLC, State College, Pennsylvania, USA) software with default settings was used for peak identification at recommended threshold intensity. The determination of the fragment size was based on the GS600 size standard. Each peak was treated as an allele at a codominant locus, and the genotype of each individual/pool at each locus was recorded. Polymorphic information content (PIC) (Botstein et al., 1980) for each marker across all accessions was determined using PowerMarker version 3.25 (Liu and Muse, 2005). Arlequin version 3.5.2.2 (Excoffier and Lischer, 2010) was used for the analysis of molecular variance (AMOVA). The pairwise *F*_*ST*_ output in an XML format was used to generate a graph through the application of a series of R scripts within Rcmd (i.e., a console version of the R statistical package) triggered through the Rcmd command button added to Arlequin version 3.5.2.2 toolbar. The Numerical Taxonomy System (NTSYS) statistical program version 2.1 (Exeter Software, New York, USA) was used to calculate Nei's standard genetic distance (GD) as described by Rohlf (2002). These GD data were used for the unweighted pair group method with arithmetic mean (UPGMA) and neighbor-joining (NJ)-based cluster analyses using the MEGA7 program (Kumar et al., 2016). The Nei's standard GD-based principal coordinate analysis (PCoA) was performed to further display the genetic relationship between the finger millet accessions using GeneAlEx 6.41 (Peakall and Smouse, 2006). The allele data set obtained from the polymorphic loci was used to identify the genetic populations for 29 indiv-accessions using STRUCTURE software version 2.3.4 (Pritchard et al., 2000). The admixture model was adopted with 100,000 burn-in periods and 200,000 Markov chain Monte Carlo (MCMC) chain iterations. To find the optimum number of clusters, a *K*-value was set from 2 to 10 with 10 independent runs. STRUCTURESELECTOR (Li and Liu, 2018) was used to visualize the threshold *K*-value (i.e., number of clusters) based on different approaches, the median of medians (MedMedK), median means (MedMeaK), maximum of medians (MaxMedK), and maximum of means (MaxMeaK) (Puechmaille, 2016). After the optimum *K*-value was determined, the CLUMPACK beta version (Kopelman et al., 2015) was used to display the graphical representation of the population structure.

## Results

### The Newly Developed EST-SSR Markers and Genetic Diversity

Thirteen new EST-SSR markers were developed in this study. The list of these EST-SSRs is given in Supplementary Table 2 along with information such as repeat motif, SSR position, the position of forward primer (start), the position of reverse primer (end), expected product size, forward and reverse primer sequences, and the full corresponding EST sequences. Six of these markers are trinucleotide repeat whereas the remaining seven are dinucleotide repeat SSRs (Supplementary Table 2). A comparative analysis of the EST sequences containing the 13 EST-SSRs against annotated genomes of Poaceae species available at https://phytozome.jgi.doe.gov/pz/portal.html (Phytozome version 12.1) showed that, on the one hand, the SSRs of *Elco-33, Elco-37, Elco-39*, and *Elco-41* are located within the coding sequences. On the other hand, the SSRs of *Elco-27* and *Elco-40* are located in the 5′UTR whereas those of *Elco-42, Elco-43*, and *Elco-48* are located in the 3′UTR (Supplementary Table 2). For example, the SSR of *Elco-27* is located 13 nucleotides upstream of the start codon of the aquaporin gene. However, there was no sufficient sequence information to determine the SSR location within the corresponding genes for *Elco-14, Elco-35, Elco-45*, and *Elco-47*. Among the 13 EST-SSRs, 6 (i.e., *Elco-14, Elco-35, Elco-41, Elco-43, Elco-45*, and *Elco-47*) were monomorphic across the 60 accessions studied. The remaining seven were polymorphic and used for the analyses of population genetics together with the three polymorphic genomic SSRs (Supplementary Table 2). The three genomic SSRs (i.e., UPEG24, UPEG27, and UPEG66) were selected from those used in the study by Dida et al. (2007).

**Figure 1:**
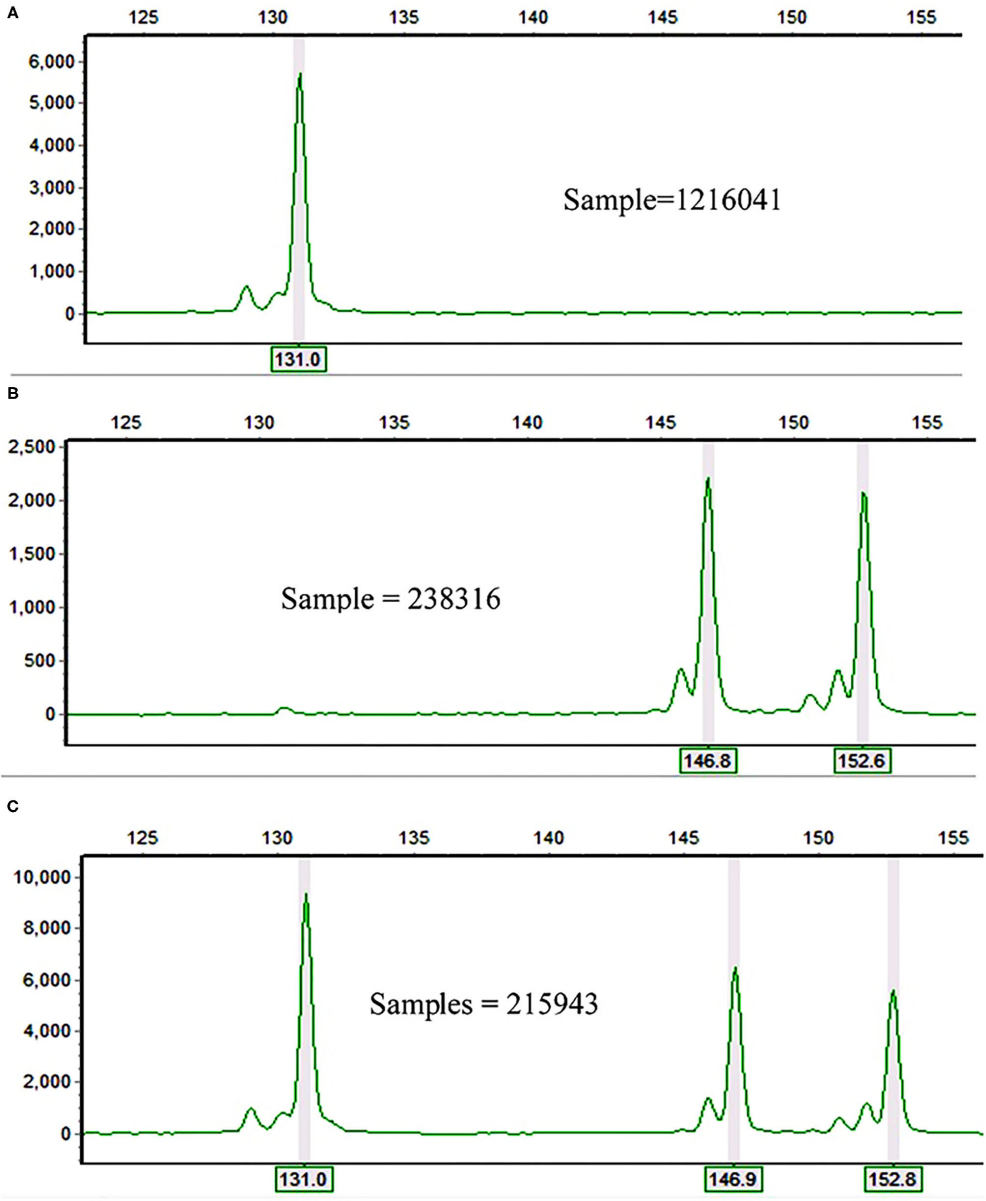
Electrophoretograms of three finger millet samples at *Elco-27* locus showing **(A)** homozygosity for the same allele (131 bp) in both **(A,B)** genomes; **(B)** either heterozygosity for the two alleles (147 and 153 bp) in both **(A,B)** genomes or homozygosity for 147 bp allele in one genome and for 153 bp in the other genome; and **(C)** homozygosity for 131 bp allele in one genome and heterozygosity for the two alleles (147 and 153 bp) in the other genome.

In total, 26 alleles were recorded across the 10 polymorphic loci and 60 accessions of finger millet. The observed number of alleles (*Na*) per locus ranged from three (in *Elco-37* and *Elco-42*) to nine (*Elco-39*) with an average value of 5.62 (Table 1). The polymorphic SSR loci accurately followed their repeat motif patterns. For example, locus *Elco-27* is a dinucleotide repeat SSR and had 131, 147, and 153 bp alleles (Figure 1) whereas the trinucleotide repeat locus *Elco-33* had 158, 161, and 172 bp alleles. The allele pattern of the three samples in Figures 1A–C is an example that demonstrates finger millet is a tetraploid species.

**Table 1:** Gene diversity estimates of each indiv-accession and its mean values (row 2–34) and number of alleles (*Na*), observed heterozygosity (*H*_*O*_), within-population gene diversity (*H*_*S*_), total gene diversity (*H*_*T*_), and polymorphic information content (PIC) of each polymorphic locus across the accessions (row 35–40).

**Acc**.	***Elco27***	***Elco33***	***Elco37***	***Elco39***	***Elco40***	***Elco42***	***Elco48***	**UPEG24**	**UPEG27**	**UPEG66**	**Mean**
215908	0.68	0.18	0.54	0.71	0.54	0.54	0.00	0.71	0.00	0.63	0.50
215929	0.8	0.1	0.6	0.8	0.6	0.6	0.00	0.71	0.57	0.53	0.59
215930	0.75	0.22	0.55	0.67	0.55	0.55	0.00	0.75	0.57	0.64	0.58
215932	0.75	0.25	0.57	0.67	0.57	0.57	0.00	0.75	0.67	0.76	0.62
215943	0.8	0.4	0.6	0.73	0.6	0.6	0.00	0.75	0.67	0.80	0.50
237443	0.75	0.28	0.57	0.57	0.57	0.57	0.00	1.00	1.00	0.80	0.68
243639	0.72	0.35	0.55	0.69	0.55	0.55	0.00	0.80	0.60	0.73	0.62
243640	0.71	0.41	0.55	0.72	0.55	0.55	0.00	0.80	0.60	0.67	0.62
216041	0.54	0.32	0.54	0.69	0.54	0.54	0.00	0.80	0.67	0.67	0.59
216042	0.75	0.01	0.42	0.71	0.57	0.57	0.00	1.00	0.00	0.68	0.52
216046	0.8	0.01	0.6	0.8	0.6	0.6	0.00	0.80	0.60	0.83	0.63
216054	0.72	0.37	0.53	0.64	0.53	0.55	0.00	0.80	0.60	0.61	0.44
237969	0.72	0.3	0.54	0.73	0.54	0.54	0.00	1.00	0.00	0.71	0.56
237971	0.71	0.14	0.53	0.71	0.53	0.53	0.00	1.00	0.00	0.71	0.54
245088	0.71	0.28	0	0.69	0.53	0.53	0.00	1.00	0.00	0.71	0.49
245092	0.71	0.13	0.53	0.7	0.53	0.53	0.00	0.71	0.53	0.67	0.56
238316	0.76	0.01	0.57	0.7	0.57	0.57	0.00	1.00	0.67	0.80	0.63
238317	0.75	0.2	0.55	0.67	0.55	0.55	0.00	0.66	1.00	0.73	0.63
238321	0.72	0.43	0.54	0.57	0.54	0.53	0.00	0.66	0.50	0.72	0.58
242612	0.68	0.01	0.55	0.53	0.55	0.55	0.00	0.80	0.50	0.75	0.55
242614	0.65	0.28	0.53	0.62	0.46	0.53	0.00	0.73	0.53	0.71	0.56
242621	0.73	0.35	0.55	0.67	0.53	0.55	0.00	0.70	0.6	0.63	0.59
242622	0.75	0.01	0.42	0.6	0.57	0.57	0.25	1.00	1.00	0.72	0.63
242623	0.72	0.15	0.51	0.71	0.53	0.53	0.15	0.83	0.67	0.67	0.59
Adis-01	0.71	0.38	0.55	0.67	0.53	0.55	0.00	0.80	0.53	0.71	0.60
Axum	0.68	0.26	0.53	0.6	0.53	0.53	0.00	0.73	0.55	0.69	0.57
Bako-09	0.71	0.36	0.55	0.68	0.55	0.55	0.00	0.75	0.55	0.68	0.60
Bareda	0.7	0.01	0.53	0.6	0.53	0.53	0.00	0.75	0.57	0.7	0.55
Boneya	0.75	0.22	0.55	0.6	0.55	0.55	0.00	0.75	0.53	0.75	0.58
Amhara	0.75	0.27	0.57	0.70	0.57	0.57	0.00	0.78	0.59	0.70	0.59
Oromia	0.71	0.20	0.46	0.71	0.55	0.55	0.00	0.89	0.30	0.70	0.54
Tigray	0.72	0.18	0.53	0.63	0.54	0.55	0.05	0.80	0.68	0.72	0.59
Cultivars	0.71	0.25	0.54	0.63	0.54	0.54	0.00	0.76	0.55	0.71	0.58
*Na*	7	5	3	9	4	3	4	7	8	5	5.62
*H_*o*_*	0.80	0.44	0.66	0.77	0.68	0.68	0.30	0.81	0.60	0.75	0.65
*H_*S*_*	0.13	0.07	0.04	0.12	0.03	0.02	0.004	0.11	0.12	0.16	0.08
*H_*T*_*	0.16	0.08	0.05	0.15	0.04	0.02	0.004	0.03	0.14	0.19	0.09
*G_*ST*_*	0.23	0.10	0.27	0.20	0.21	0.02	0.00	0.20	0.19	0.18	0.12
*PIC*	0.53	0.23	0.31	0.65	0.21	0.17	0.045	0.68	0.67	0.71	0.51

The total gene diversity (*H*_*T*_) of each locus across all populations ranged from 0.004 in locus *Elco-48* to 0.19 in locus *UPEG-66* with an average value of 0.09. Among the 29 indiv-accessions, *Elco-48* was polymorphic only in two accessions (242622 and 243623), which were originally collected from western Tigray. Similarly, two accessions that were polymorphic only for *Elco-48* (accessions 215908 and 215929) were identified among the pooled accessions. These accessions were originally collected from Agew Awi and Gojam, respectively. The within-population gene diversity (*H*_*S*_) of each locus varied from 0.004 to 0.16 with an average value of 0.08. The observed heterozygosity (*H*_*O*_) for each locus varied from 0.30 (*Elco-48*) to 0.81 (*UPEG-24*). The estimates of population differentiation (*G*_*ST*_) at each locus varied from 0 (*Elco-48*) to 0.27 (*Elco-37*) with a mean of 0.12. Similarly, the lowest (0.045) and highest (0.71) PIC values were recorded for *Elco-48* and *UPEG-66*, respectively, with a mean of 0.51 (Table 1).

The mean gene diversity estimates of each accession across the 10 polymorphic loci showed narrow variation ranging from 0.44 (accession 216054) to 0.68 (accession 237443) (Table 1). Similarly, a narrow range of variation was recorded at the level of regional states ranging from 0.54 (Oromia) to 0.59 (Amhara and Tigray). Interestingly, the average gene diversity of the landrace accessions (0.57) was similar to that of the cultivars (0.58).

### Genetic Variation Within and Among Accessions

The AMOVA based on allele frequency of the 60 accessions (combined data set of indiv-accessions and pool-accessions) was conducted to determine the genetic differentiation at accession and higher hierarchical levels (Table 2). AMOVA that was conducted by grouping the accessions according to their geographic regions of origin and without grouping revealed a highly significant variation among accessions and regions as well as within accessions (Table 2; *P* < 0.0001). Of the total genetic variation, 18.8% accounted for the differentiation among the accessions (*F*_*ST*_ = 0.18) whereas 81.2% was varied within the accessions. Furthermore, when the accessions were pooled to their respective regions, variation among groups (regions) and among accessions within groups were 6.9 and 13.2%, respectively.

**Table 2:** Analysis of molecular variance (AMOVA) of 60 finger millet accessions (31 indiv-accessions plus 29 pool-accessions) without grouping and by grouping them according to their region of origin.

**SV**	**DF**	**SS**	**VC**	**PV**	**FI**	***P*-value**
**(A) Without grouping the accessions**						
Among accessions	59	145.84	Va = 0.37	18.77	F_ST_ = 0.18	Va and F_ST_ < 0.001
Within accessions	406	408.23	Vb = 1.60	81.23		
Total	465	554.08	1.97			
**(B) By grouping the accessions according to their region of origin**						
Among regions	4	42.03	Va = 0.13	6.87	F_CT_ = 0.07	Va and F_CT_ < 0.001
AAWR	55	103.81	Vb = 0.26	13.18	F_SC_ = 0.14	Vb and F_SC_ < 0.001
Within accessions	406	408.23	Vc = 1.60	79.94	F_ST_ = 0.20	Vc and F_ST_ < 0.001
Total	465	554.08	2.00			

*SV, source of variation; DF, degrees of freedom; SS, sum of squares; VC, variance component; PV, percentage of variation; FI, fixation index; AAWR, among accessions within regions*.

### GD, Population Differentiation, Cluster, and Population Structure Analyses

**Figure 2:**
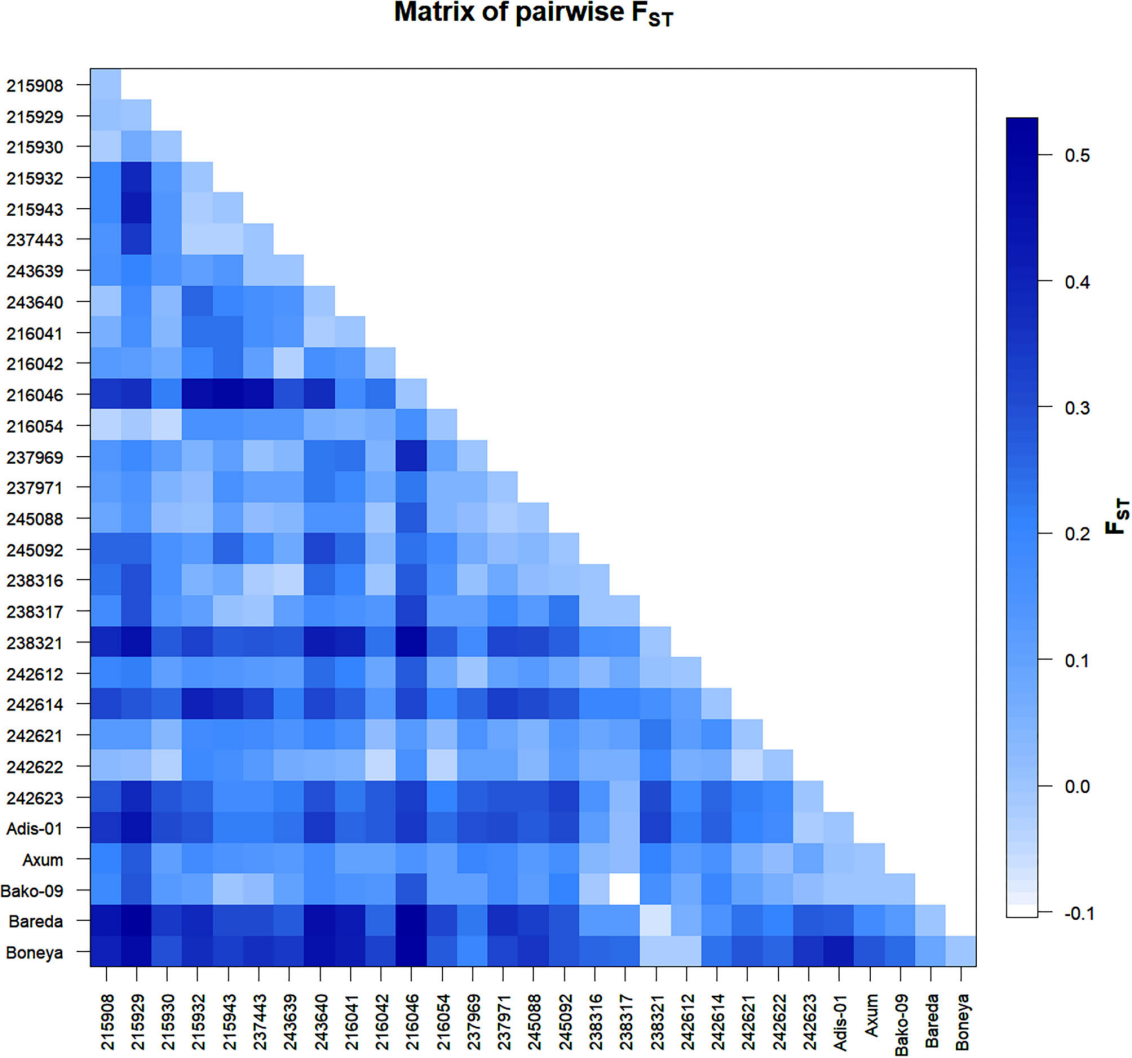
Graphical representation of pairwise *F*_*ST*_ between 29 indiv-accessions of finger millet.

The Nei's pairwise GD among the 29 indiv-accessions ranged from 0 indicating high genetic similarity (215908 vs. 243640; 245088 vs. 215932; 238317 vs. 237443, and Bako-09 vs. 215943) to slightly over 0.5 showing low genetic similarity (Bareda vs. 215929, Bareda vs. 216046, and Boneya vs. 216046). Accession 216046 from Oromia is the most genetically distinct accession with a mean GD of 0.32 from the other 28 accessions. The highest GD between accessions within a region was 0.42 for Amhara (215929 vs. 215943), 0.38 for Oromia (216046 vs. 237969), and 0.31 for Tigray (238321 vs. 242623). The highest GD within the cultivar group (0.41) was recorded for Boneya vs. Addis-01 cultivar (Table 3). The mean GD between accessions within Amhara, Oromia, Tigray, and cultivar groups were 0.13, 0.10, 0.14, and 0.15, respectively. Among regions, the mean GD of 0.15, 0.18, and 0.20 were recorded for Amhara vs. Oromia, Oromia vs. Tigray, and Amhara vs. Tigray, respectively. Against the cultivars, the mean GDs of 0.27, 0.25, and 0.14 were recorded for Amhara, Oromia, and Tigray regions. The pairwise *F*_*ST*_ values demonstrated in Figure 2 are in agreement with the overall significant differentiation (*F*_*ST*_ = 0.18; *P* < 0.0001) among all accessions studied.

**Table 3:** Pairwise Nei's standard genetic distance between the 29 finger millet indiv-accessions calculated based on the data from 10 simple sequence repeat (SSR) loci.

**Accession**	**1**	**2**	**3**	**4**	**5**	**6**	**7**	**8**	**9**	**10**	**11**	**12**	**13**	**14**	**15**	**16**	**17**	**18**	**19**	**20**	**21**	**22**	**23**	**24**	**25**	**26**	**27**	**28**	**29**	
215908	1	**0.19**																												
215929	2	0.01	**0.25**																											
215930	3	0.02	0.07	**0.14**																										
215932	4	0.19	0.39	0.13	**0.19**																									
215943	5	0.19	0.42	0.14	0.02	**0.19**																								
237443	6	0.14	0.34	0.14	0.03	0.03	**0.15**																							
243639	7	0.15	0.21	0.15	0.11	0.14	0.00	**0.15**																						
243640	8	0.00	0.18	0.03	0.26	0.20	0.16	0.15	**0.22**																					
216041	9	0.06	0.17	0.04	0.24	0.23	0.17	0.14	0.02	**0.19**																				
216042	10	0.13	0.11	0.07	0.18	0.23	0.11	0.03	0.17	0.14	**0.13**																			
216046	11	0.35	0.37	0.22	0.46	0.50	0.46	0.30	0.38	0.18	0.24	**0.32**																		
216054	12	0.04	0.01	0.05	0.15	0.16	0.13	0.13	0.06	0.05	0.07	0.17	**0.13**																	
237969	13	0.14	0.19	0.12	0.05	0.11	0.01	0.04	0.23	0.23	0.05	0.38	0.09	**0.14**																
237971	14	0.11	0.15	0.05	0.02	0.16	0.11	0.11	0.23	0.19	0.08	0.23	0.05	0.04	**0.16**															
245088	15	0.08	0.14	0.02	0.00	0.11	0.02	0.04	0.15	0.15	0.00	0.28	0.05	0.02	0.02	**0.13**														
245092	16	0.26	0.26	0.16	0.12	0.25	0.17	0.08	0.31	0.25	0.03	0.23	0.18	0.07	0.02	0.04	**0.18**													
238316	17	0.24	0.30	0.16	0.05	0.07	0.02	0.04	0.25	0.20	0.00	0.28	0.15	0.01	0.08	0.02	0.01	**0.11**												
238317	18	0.18	0.30	0.14	0.11	0.01	0.00	0.11	0.18	0.16	0.14	0.33	0.11	0.10	0.19	0.13	0.22	0.00	**0.13**											
238321	19	0.38	0.45	0.28	0.33	0.27	0.29	0.28	0.41	0.39	0.24	0.49	0.27	0.18	0.32	0.30	0.27	0.17	0.16	**0.26**										
242612	20	0.19	0.21	0.11	0.15	0.14	0.13	0.12	0.25	0.21	0.09	0.28	0.08	0.00	0.10	0.13	0.08	0.03	0.08	0.01	**0.12**									
242614	21	0.32	0.29	0.26	0.41	0.37	0.33	0.22	0.32	0.27	0.14	0.31	0.20	0.25	0.34	0.31	0.28	0.19	0.20	0.16	0.10	**0.24**								
242621	22	0.13	0.13	0.04	0.18	0.19	0.18	0.15	0.20	0.15	0.02	0.12	0.03	0.15	0.09	0.04	0.14	0.08	0.10	0.22	0.11	0.17	**0.13**							
242622	23	0.03	0.02	0.03	0.19	0.16	0.13	0.07	0.06	0.05	0.05	0.15	0.04	0.10	0.10	0.03	0.13	0.06	0.06	0.19	0.06	0.07	0.05	**0.10**						
242623	24	0.29	0.38	0.28	0.26	0.18	0.18	0.21	0.29	0.23	0.27	0.34	0.21	0.27	0.29	0.29	0.32	0.15	0.03	0.31	0.19	0.26	0.21	0.19	**0.23**					
Adis-01	25	0.36	0.44	0.31	0.29	0.22	0.21	0.23	0.34	0.25	0.27	0.35	0.25	0.30	0.31	0.28	0.31	0.11	0.02	0.33	0.22	0.26	0.20	0.18	0.02	**0.24**				
Axum	26	0.21	0.27	0.11	0.17	0.15	0.13	0.13	0.17	0.09	0.10	0.15	0.11	0.20	0.17	0.13	0.16	0.04	0.01	0.21	0.12	0.16	0.05	0.02	0.09	0.01	**0.13**			
Bako-09	27	0.18	0.29	0.13	0.10	0.00	0.01	0.10	0.17	0.15	0.14	0.28	0.10	0.11	0.17	0.12	0.20	0.01	0.10	0.17	0.08	0.20	0.10	0.06	0.02	0.00	0.00	**0.12**		
Bared	28	0.45	0.52	0.34	0.38	0.30	0.31	0.28	0.46	0.42	0.26	0.53	0.32	0.22	0.37	0.34	0.29	0.13	0.13	0.07	0.06	0.15	0.24	0.21	0.27	0.26	0.18	0.13	**0.28**	
Boneya	29	0.41	0.48	0.29	0.37	0.34	0.37	0.35	0.46	0.42	0.32	0.52	0.28	0.20	0.32	0.35	0.29	0.26	0.25	0.02	0.02	0.23	0.28	0.26	0.36	0.41	0.29	0.25	0.09	**0.30**

*Bold diagonal values, mean Nei's standard genetic distance of each accession against all other accessions. Accessions with codes 1–8, 9–16, and 17–24 were collected from Amhara, Oromia, and Tigray, respectively, whereas the last five accessions (with codes 25–29) are the improved cultivars*.

**Figure 3:**
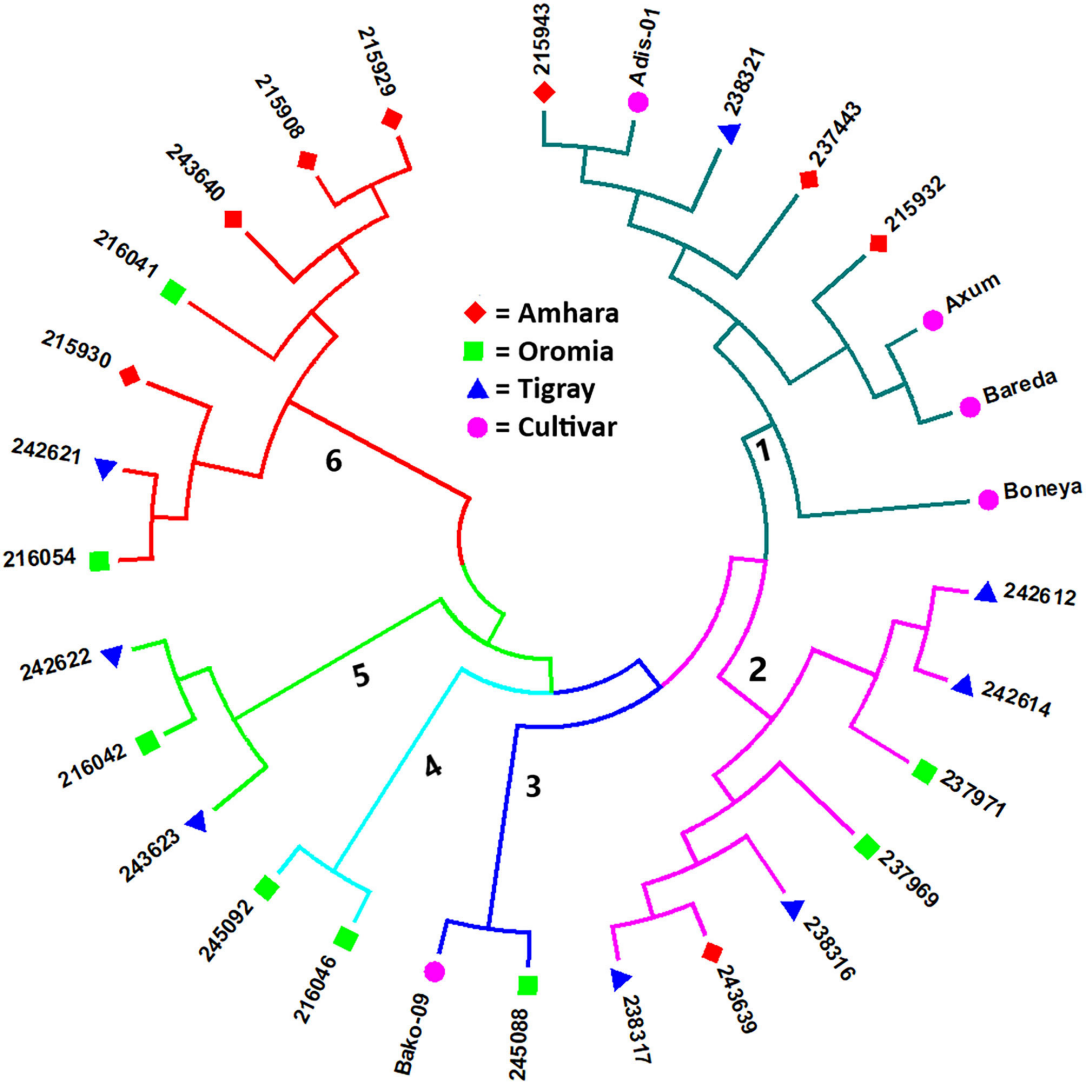
Nei's standard GD-based NJ tree showing the clustering pattern of the 29 indiv-accessions of finger millet. Accessions sharing a symbol with the same shape and color belong to the same group (regions or cultivar).

The Nei's standard GD-based NJ dendrogram revealed six clusters with each cluster comprising accessions from different groups (i.e., geographical regions and cultivar) except Cluster-4, which comprised only two accessions from Oromia. Cluster-1 was the largest comprising eight accessions composed of three accessions from Amhara, one accession from Tigray, and four cultivars. Cluster-2 was composed of four accessions from Tigray, two from Oromia, and one from Amhara. Cluster-3 comprised one accession from Oromia and a cultivar. Cluster-5 comprised two accessions from Tigray and one from Oromia, whereas four accessions from Amhara, two from Oromia, and one from Tigray were placed in Cluster-6 (Figure 3).

**Figure 4:**
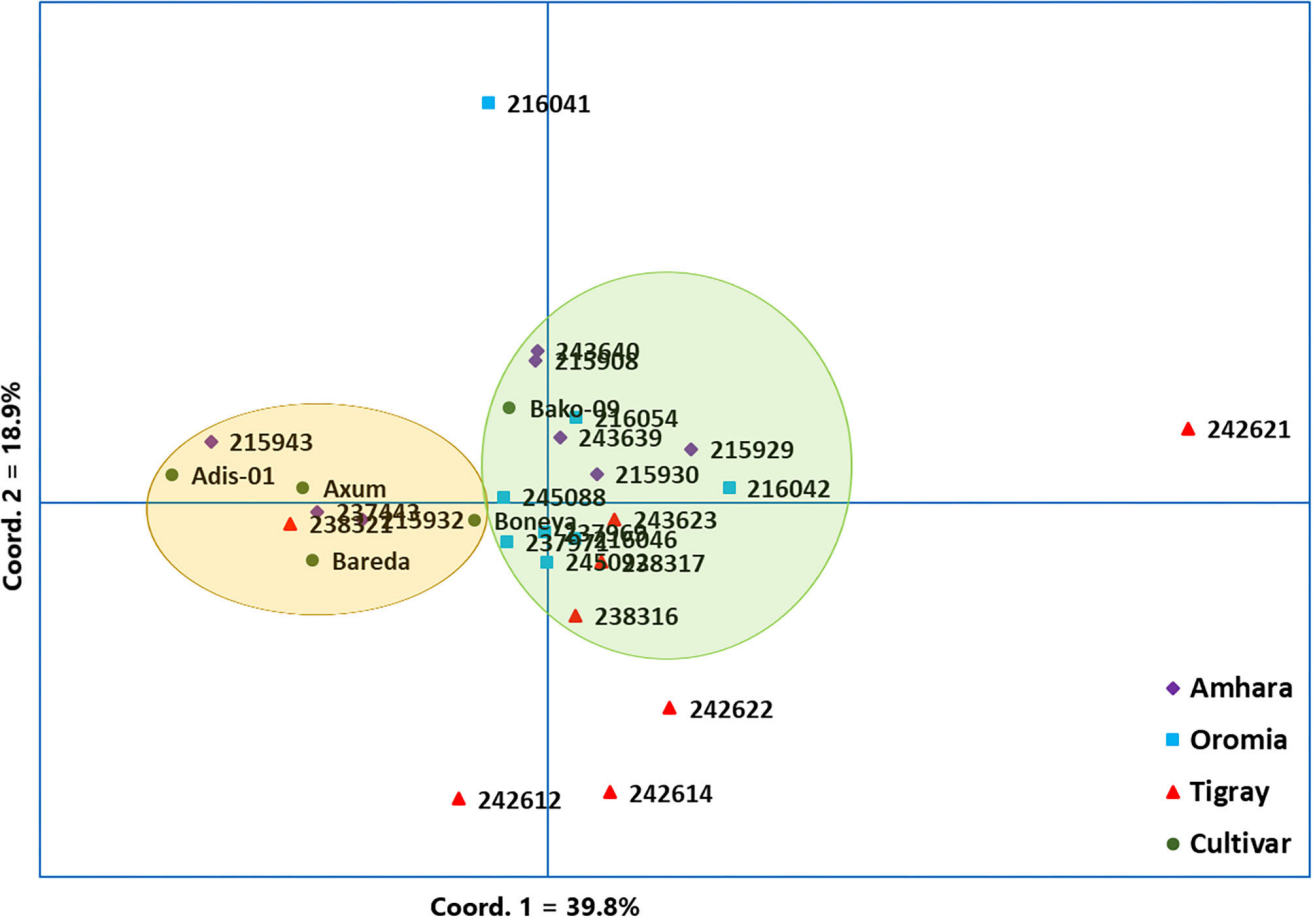
Principal coordinate analysis (PCoA) based on 10 simple sequence repeat (SSR) markers, depicting the genetic relationship between the 29 finger millet indiv-accessions. Accessions originated from different regions and the improved cultivars were represented by different symbols.

The PCoA was used to visualize the differentiation among the 29 indiv-accessions (Figure 4). The analysis revealed that the first and second coordinates accounted for 39.8% and 18.9% of the total variation, respectively. Hence, the two coordinates together explained 58.7% of the total variation. Along the first coordinate, a group comprising eight accessions (i.e., three accessions from Amhara, one from Tigray, and four from the improved cultivar group) was separated (highlighted in light yellow) from the major group (highlighted in light green) whereas an accession from Tigray (242621) was an outlier. Similarly, three accessions were separately grouped along the second coordinate. An outlier accession (216041) was also observed along the second coordinate (Figure 4). Overall, the PCoA revealed the absence of a clear clustering pattern of the accessions according to their geographic regions of origin, similar to the results obtained through the cluster analysis (Figure 3).

**Figure 5:**
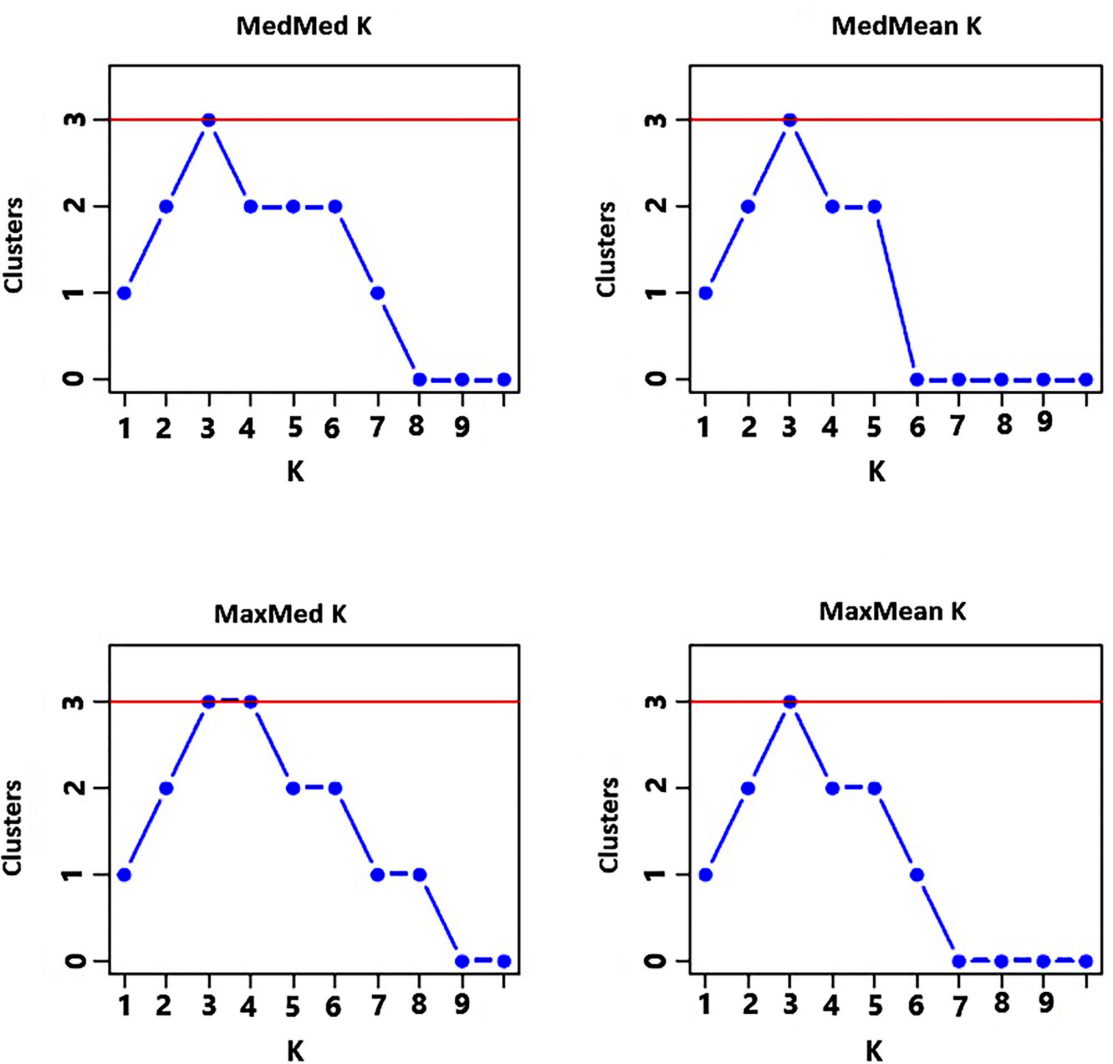
Graphs displaying an optimum of three genetic clusters representing the genotypes of the 29 indiv-accessions based on the approach of Puechmaille (2016) of determining the optimum number of clusters.

**Figure 6:**
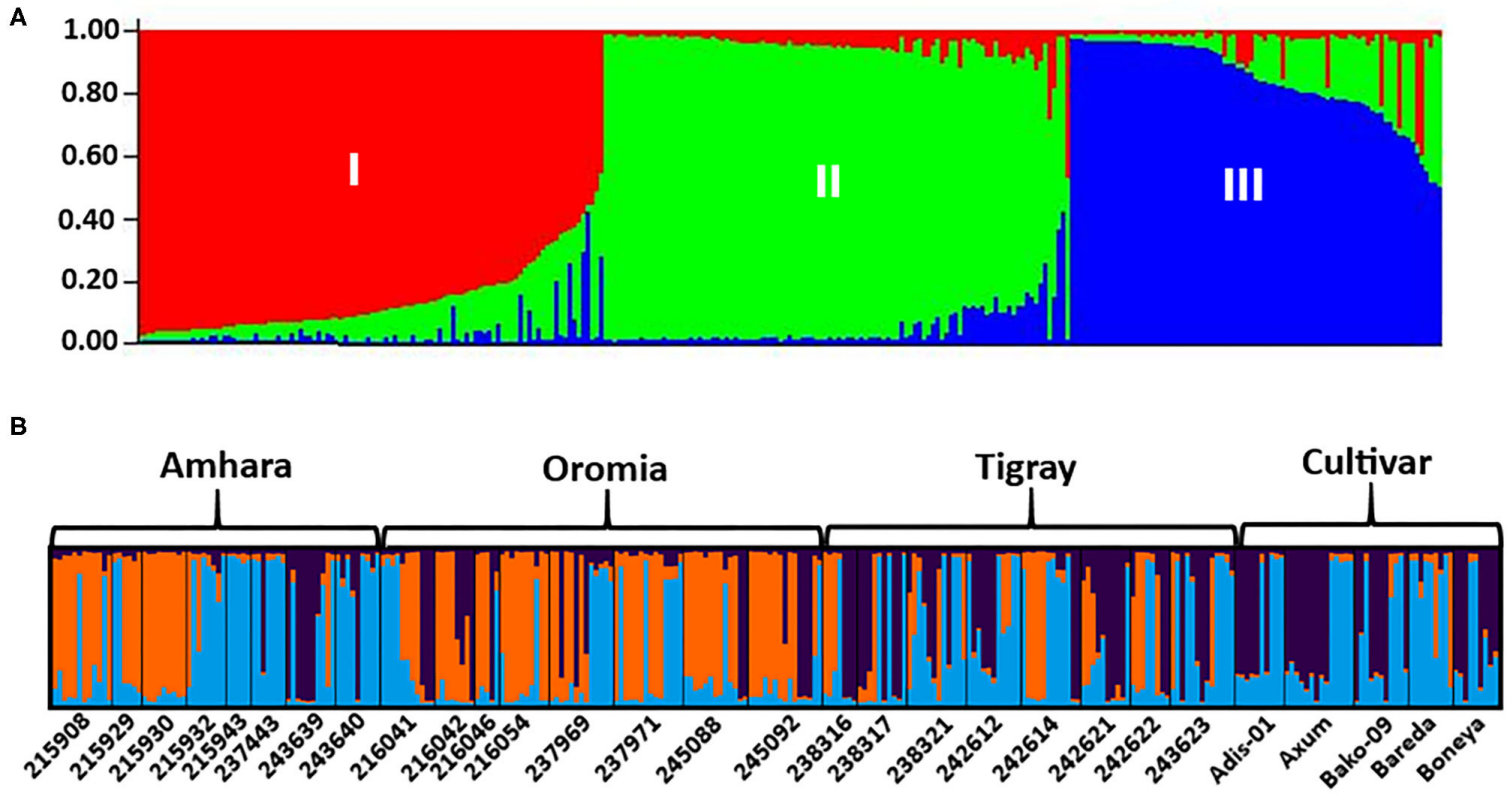
The population genetic structure of 29 indiv-accessions of finger millet for *K* = 3. Each color represents a different cluster, and the different colors of each genotype represent membership in the different genetic populations. **(A)** Graphical representation of individual genotypes arranged according to the level of their membership in different clusters; **(B)** graphical display of the genetic structure of each accession (8, 8, 8, and 5 accessions representing Amhara, Oromia, Tigray, and cultivars, respectively).

The analysis of the population structure of the 29 indiv-accessions was conducted based on the data from the 10 polymorphic loci using STRUCTURE software. For this analysis, the approach of Puechmaille (2016) was used, which revealed that the optimum *K* = 3 (i.e., MedMeaK, MaxMeaK, MedMedK, and MaxMedK = 3), indicating that the individual genotypes of the 29 indiv-accessions most likely came from three genetic populations (Figure 5). The STRUCTURE output at *K* = 3 displayed partial membership of all indiv-accessions to more than one cluster (Figure 6), suggesting a strong population admixture.

## Discussion

### The Newly Developed EST-SSR Markers and Genetic Diversity

In general, compared with other markers, the EST-SSR markers are appropriate and provide useful genetic information in diversity studies due to their high transferability across species and association with differentially expressed genes (Gupta et al., 2003; Saha et al., 2004; Varshney et al., 2005). In this study, most of the newly developed EST-SSR markers showed high transferability across different crops and they are linked to the coding regions of different genes. For example, the EST-SSR of *Elco-27* is linked (only 13 nucleotides upstream of the start codon, data not shown) to the aquaporin gene in maize and rice. The aquaporin gene is responsible for water stress-induced chilling tolerance (Li et al., 2000). The EST-SSR of *Elco-42* is linked to a gene coding for a chemocyanin-like protein that plays a positive role in a response to salinity stress and stripe rust in wheat (Feng et al., 2013). In addition to marking aquaporin coding genes and chemocyanin-like protein genes, the newly developed EST-SSR markers are also important for species identification, gene identification, and conservation of genetic material. In this study, 6 of the 13 newly developed EST-SSRs were monomorphic, and those that are monomorphic within *E. coracana* can probably serve as species-specific markers, differentiating it from other *Eleusine* species.

Among the seven new polymorphic EST-SSRs, three of them were located in the CDS (coding sequences) whereas four were located in either 3′UTR or 5′UTR. Interestingly, those in the CDS were more informative (mean PIC = 0.40) than the others (mean PIC = 0.24). The three SSRs in the CDS were trinucleotide repeat SSRs and hence do not cause frameshift mutations, which might have contributed to their higher polymorphism. Among the new EST-SSRs, *Elco-27* and *Elco-39* were the most informative with PIC values of 0.53 and 0.65, respectively. Hence, they need to be prioritized for use in population genetics studies. The level of *H*_*O*_ obtained in this study was similar to that of Bharathi (2011) and Babu et al. (2018) who reported values ranging from 0.20 to 0.85 and from 0 to 0.88, respectively. Given that finger millet is a predominately self-pollinating species, such a relatively high level of heterozygosity is not expected. The possible explanation for high heterozygosity is the fact that it is an amphidiploid, and the SSR loci used are most likely found in both A and B genomes. Hence, although the heterozygosity within a genome can be very low due to self-fertilization, *H*_*O*_ could be high due to the dominance of different alleles in the A and B genomes. Among the three *UPEG* SSR loci, *UPEG24* was mapped to chromosome 3B (Dida et al., 2007). If the locus is specific to the B genome, the high heterozygosity at this locus may suggest that the locus is under selection that favors heterozygosity. This locus was associated with productive tiller number whereas *UGEP66* was associated with grain yield and thousand-grain weight (Lule et al., 2018). Hence, breeders can use these markers in their marker-aided breeding programs to increase the productivity of finger millet. It is interesting to investigate if any of the alleles identified in this study at these loci are associated with desirable characteristics of these traits.

The level of genetic variation in populations can be estimated by different statistical parameters, such as *H*_*O*_, *H*_*T*_, *H*_*S*_, percentage of polymorphic loci (PPL), and Nei's gene diversity (*H*_*E*_). The PIC of an SSR locus indicates the extent of its usefulness in the determination of population genetic diversity. Similar to other population genetics parameters, the PIC of a locus is dependent on the number and frequency of alleles at that locus. The higher the PIC value of a locus, the more informative it is. In this study, a broad range of PIC values (0.05–0.71) was obtained. Interestingly, the average PIC of the seven EST-SSRs (0.31) was lower than the mean PIC of the three previously published SSRs (0.69) (Dida et al., 2008), which is expected as EST-SSRs are more conserved than genomic SSRs in general. The mean PIC values of these three primers in this study (0.69) are lower than previously reported (0.88) (Lule et al., 2018). The higher values reported in the study by Lule et al. (2018) can be explained by the more diverse germplasm used which led to the recording of a larger number of alleles (sum = 61) than those recorded in this study (sum = 20). The gene diversity estimates of the accessions varied from 0.44 (accession 216054 from Oromia) to 0.68 (accession 237443 from Amhara) with an overall mean of 0.58. To identify the local genetic diversity hot spots for finger millet, further study needs to be conducted in the localities where accessions with the above-average gene diversity estimates were obtained. Interestingly, the average gene diversity of the landrace accessions and that of the cultivars were similar, implying that breeding activities did not have a significant effect on the genetic diversity of the crop.

### Genetic Variation Within and Among Populations

The AMOVA revealed significant genetic variations both among and within the finger millet accessions. A higher percentage of variation (81.23%) was detected within accessions compared with that of among accessions (18.77%), which is in line with the fact that finger millet is a predominantly self-pollinating species. Babu et al. (2014) reported 73% within-population and 27% among-population variation in the study conducted on 190 accessions of finger millet using 75 genic SSR markers. Similarly, Lule et al. (2018) found 69.52% within-population and 30.48% among-population variation in 138 finger millet accessions using SSR markers. Furthermore, research by Pandian et al. (2018) on 83 finger millet genotypes using 43 genic SSR markers revealed 77% within-population and 23% among-population genetic variation. The significantly higher within-population variation than the among-population variation in finger millet could be partly explained by the fact that farmers select finger millet genotypes based on different criteria, such as good performance in grain yield and tolerance to various biotic and abiotic stresses. Only the farmer-selected individuals contribute seeds to the next generation, and hence, the selection of genetically diverse genotypes leads to high genetic diversity within populations. The amphidiploid nature of finger millet is also another contributor to genetic variation within populations. The lower genetic differentiation among populations compared with the variation within populations, which is in agreement with other published studies (Babu et al., 2014; Lule et al., 2018; Pandian et al., 2018), could be explained by strong gene flow through the market channel-based seed exchange and the use of the same improved cultivars among neighboring regions in Ethiopia.

### Genetic Relationship and Population Structure Analysis

The NJ cluster analysis of the 29 indiv-accessions revealed a weak clustering pattern of the accessions according to the geographic regions of origin of the landrace accessions, indicating that the genetic makeup of the accessions does not have a strong correlation with their geographic origin. The clustering together of accessions from different regions, as well as the placement of accessions from geographically close areas in different clusters in this study, agreed with the results of previous studies. For instance, Pandian et al. (2018) reported 3 clusters for 83 accessions of finger millet using 43 genic SSR markers, Lule et al. (2018) reported 3 clusters for 138 accessions evaluated using 20 SSR markers, and Ramakrishnan et al. (2016) reported 3 clusters for 128 Indian finger millet accessions evaluated using 87 genomic SSR markers. Given that the first two principal coordinates of the PCoA biplot (Figure 5) explained only 58.7% of the total variation, a discrepancy between the clustering patterns of the accessions displayed in the cluster analysis and PCoA is expected. However, the PCoA analysis also revealed a weak clustering of the accessions according to their geographic origin, in agreement with the result of the cluster analysis. The results suggest a strong gene flow between the geographic regions. The fact that most of the cultivars were clustered closely together suggests that they might have been developed based on genetically similar germplasm and/or selected for similar traits during the breeding process. However, Boneya and Adis-01 were described as the best cultivar in terms of grain yield and stability, tolerance to disease, and also other agronomic performance (Negash et al., 2019).

The population structure analysis distinguished three genetic groups (*K* = 3), which were not based on the geographical origin of the accessions. Some individual genotypes from different accessions showed highly similar genetic profiles and hence have a close relationship. Previous studies, using SSR markers on different finger millet accessions, also produced three genetic clusters having week grouping based on their geographic origin (Dida et al., 2007; Ramakrishnan et al., 2016; Lule et al., 2018; Pandian et al., 2018). All accessions and individual samples analyzed had alleles from the three genetic populations indicating strong gene flow that led to poor genetic differentiation among the accessions. Although there are some exceptions, there is a good general agreement between different analyses in terms of grouping the accessions. For example, the seven accessions in Cluster-6 of the NJ tree (Figure 3) were clustered closely together (highlighted in light yellow) in the PCoA biplot (Figure 4). Similarly, most accessions in Cluster-1 of the NJ tree (Figure 3) were mainly represented by the combinations of purple and blue clusters in the STRUCTURE output (Figure 6B), whereas most of the accessions in Cluster-6 of the NJ tree (Figure 3) were dominated by orange cluster in the STRUCTURE output (Figure 6B). Overall, given the result of the cluster and genetic structure analyses, there are indications of the complex genetic composition of finger millet landrace accessions, and this needs extensive study using different molecular tools, as in other crops (Thurber et al., 2013; Qiu et al., 2014).

## Conclusion

The population genetics analyses using seven newly developed EST-SSR and three genomic SSR markers revealed significant genetic variation both within and among accessions, with over 80% of the variation residing within the accessions. There was also a significant variation among regions, suggesting stronger gene flow within regions than among regions. Given the results of the cluster, PCoA, and population structure analyses, it can be concluded that the grouping of the accessions is poorly correlated with the geographical origin of finger millet grown in Ethiopia. The STRUCTURE analysis revealed that the accessions belong to three genetic populations with strong admixture in each accession. Although accessions differ in the level of their genetic diversity to some extent, the level of diversity at the regional level is quite similar. This suggests that there is no hot spot for finger millet genetic diversity and all areas where the crop is grown should receive similar attention for the conservation of its genetic resources and use in breeding programs. The genetic diversity in landrace populations was also similar to that of the cultivars, and hence, the process of breeding did not lead to the loss of genetic diversity. This study contributed novel EST-SSR markers for their various applications, such as population genetics analyses and association studies, and hence, it promotes the efforts of molecular breeding in finger millet. The EST-SSRs that were monomorphic in this study should be tested in other finger millet genetic resources found in Ethiopia and beyond, to detect new alleles with potentially useful applications.

## Data Availability Statement

The original contributions presented in the study are included in the article/Supplementary Material, further inquiries can be directed to the corresponding author.

## Author Contributions

HB, TH, KT, CH, RO, and MG: conceptualization. HB, CH, and MG: methodology. HB: data collection under the guidance of MG and CH, data analysis under the guidance of MG, and writing—original draft. HB, TH, KT, CH, RO, KA, and MG: review and editing. TH, KT, CH, RO, and MG: project funding acquisition and administration. All authors have read and agreed to the final version of the manuscript.

## Funding

This study was supported by the Swedish International Development Cooperation Agency (Sida) Research and Training Grant awarded to the Addis Ababa University and the Swedish University of Agricultural Sciences (AAU-SLU Biotech; https://sida.aau.edu.et/index.php/biotechnology-phd-program/; accessed on August 20, 2021).

## Conflict of Interest

The authors declare that the research was conducted in the absence of any commercial or financial relationships that could be construed as a potential conflict of interest.

## Publisher's Note

All claims expressed in this article are solely those of the authors and do not necessarily represent those of their affiliated organizations, or those of the publisher, the editors and the reviewers. Any product that may be evaluated in this article, or claim that may be made by its manufacturer, is not guaranteed or endorsed by the publisher.
