# Supplementary material for: Novel Expressed Sequence Tag-Derived and Other Genomic Simple Sequence Repeat Markers Revealed Genetic Diversity in Ethiopian Finger Millet Landrace Populations and Cultivars
Source: Front Plant Sci. 2021 Sep 23;12:735610. doi: 10.3389/fpls.2021.735610 (PMC8495221; doi:10.3389/fpls.2021.735610)
Supplement: Supplementary file 1 [file Table_1.DOCX]

Supplementary Material

Table S1. List of 60 finger millet accessions used in this study, comprising 55 landrace accessions from four regional states in Ethiopia (with their latitude, longitude and altitude) and five cultivars.

| S.N | **Acc. No** | **Region** | **Zone** | **Latitude** | **Longitude** | **Altitude** |
| --- | --- | --- | --- | --- | --- | --- |
| 1 | 215908 | Amhara | Agew awi | 11-10-00-N | 36-52-00-E | 2250 |
| 2 | 243639 | Amhara | Agew awi | ------------ | ------------ | 2070 |
| 3 | 243641 | Amhara | Agew awi | 10-57-00-N | 36-52-00-E | 2200 |
| 4 | 215829 | Amhara | Gojam | 10-41-00-N | 37-13-00-E | 1990 |
| 5 | 215831 | Amhara | Gojam | 10-42-00-N | 37-11-00-E | 2000 |
| 6 | 215832 | Amhara | Gojam | 10-42-00-N | 37-11-00-E | 2030 |
| 7 | 215833 | Amhara | Gojam | 10-42-00-N | 37-11-00-E | 2030 |
| 8 | 215929 | Amhara | Gojam | ------------ | ------------ | --- |
| 9 | 215930 | Amhara | Gojam | ------------ | ------------ | --- |
| 10 | 215932 | Amhara | Gojam | ------------ | ------------ | --- |
| 11 | 215943 | Amhara | Gojam | ------------ | ------------ | --- |
| 12 | 215857 | Amhara | Gondar | 11-35-00-N | 37-40-00-E | --- |
| 13 | 215858 | Amhara | Gondar | 11-30-00-N | 37-35-00-E | --- |
| 14 | 215859 | Amhara | Gondar | ------------ | ------------ | --- |
| 15 | 237443 | Amhara | Wello | 12-09-00-N | 39-39-00-E | 2100 |
| 16 | 207964 | Oromia | Illubabor | 08-23-00-N | 36-17-00-E | --- |
| 17 | 245092 | Oromia | Illubabor | ------------ | ------------ | 1954 |
| 18 | 207962 | Oromia | Wellega | 09-40-00-N | 37-00-00-E | --- |
| 19 | 216027 | Oromia | Wellega | 09-09-00-N | 36-23-00-E | 2180 |
| 20 | 216028 | Oromia | Wellega | 09-09-00-N | 36-23-00-E | 2150 |
| 21 | 216031 | Oromia | Wellega | 09-12-00-N | 35-47-00-E | 1890 |
| 22 | 216032 | Oromia | Wellega | 09-12-00-N | 35-45-00-E | 1900 |
| 23 | 216038 | Oromia | Wellega | 09-24-00-N | 35-37-00-E | 2030 |
| 24 | 216041 | Oromia | Wellega | 09-32-00-N | 35-30-00-E | 1960 |
| 25 | 216042 | Oromia | Wellega | 09-32-00-N | 35-28-00-E | 1890 |
| 26 | 216046 | Oromia | Wellega | 09-40-00-N | 35-17-00-E | 1680 |
| 27 | 216054 | Oromia | Wellega | 08-57-00-N | 35-15-00-E | 1730 |
| 28 | 237969 | Oromia | Wellega | ------------ | ------------ | 1930 |
| 29 | 237971 | Oromia | Wellega | ------------ | ------------ | 2220 |
| 30 | 245088 | Oromia | Wellega | ------------ | ------------ | 2060 |
| 31 | 203326 | SNNP | Bench maji | 05-16-00-N | 37-30-00-E | 1500 |
| 32 | 203339 | SNNP | Bench maji | 05-20-67-N | 37-14-99-E | 1440 |
| 33 | 203340 | SNNP | Bench maji | 05-24-90-N | 37-14-06-E | 1500 |
| 34 | 235699 | SNNP | Bench maji | 05-16-00-N | 37-30-00-E | 1500 |

| S.N | Acc. No | Region | Zone | Latitude | Longitude | Altitude |
| --- | --- | --- | --- | --- | --- | --- |
| 35 | 241768 | SNNP | Bench maji | 05-24-90-N | 37-14-06-E | 2169 |
| 36 | 241769 | SNNP | Bench maji | 05-20-67-N | 37-14-99-E | --- |
| 37 | 203364 | SNNP | Keficho | ------------ | ------------ | 1500 |
| 38 | 240506 | SNNP | Keficho | ------------ | ------------ | --- |
| 39 | 203372 | SNNP | Kembata | ------------ | ------------ | 1440 |
| 41 | 242612 | Tigray | Debubawi | 13-59-14-N | 39-74-65-E | 2120 |
| 42 | 238313 | Tigray | Mehakelawi | 14-02-00-N | 38-04-00-E | 2110 |
| 43 | 219825 | Tigray | Mirabawi | ------------ | ------------ | --- |
| 44 | 219826 | Tigray | Mirabawi | ------------ | ------------ | --- |
| 45 | 219828 | Tigray | Mirabawi | ------------ | ------------ | --- |
| 46 | 219832 | Tigray | Mirabawi | 14-04-00-N | 38-16-00-E | 1920 |
| 47 | 242621 | Tigray | Mirabawi | 14-38-50-N | 38-17-46-E | 1770 |
| 48 | 242622 | Tigray | Mirabawi | 14-38-26-N | 38-17-50-E | 1720 |
| 49 | 242623 | Tigray | Mirabawi | 13-32-50-N | 38-20-71-E | 1590 |
| 50 | 234147 | Tigray | Misrakawi | 14-12-00-N | 39-17-00-E | 2140 |
| 51 | 237458 | Tigray | Misrakawi | 14-06-00-N | 39-30-00-E | 2100 |
| 52 | 238316 | Tigray | Misrakawi | 14-30-00-N | 39-50-00-E | 2060 |
| 53 | 238317 | Tigray | Misrakawi | 14-10-00-N | 39-52-00-E | 2090 |
| 54 | 238321 | Tigray | Misrakawi | 13-09-00-N | 39-09-00-E | 1920 |
| 55 | 242614 | Tigray | Misrakawi | 14-46-03-N | 39-50-34-E | 1970 |
| 56 | Adis-01 | Cultivar | ------------ | ------------ | ------------ | --- |
| 57 | Axum | Cultivar | ------------ | ------------ | ------------ | --- |
| 58 | Bako-9 | Cultivar | ------------ | ------------ | ------------ | --- |
| 59 | Bareda | Cultivar | ------------ | ------------ | ------------ | --- |
| 60 | Boneya | Cultivar | ------------ | ------------ | ------------ | --- |

Note: Accessions 1 to 55 are landrace populations whereas accessions 56 to 60 are cultivars released in Ethiopia. SNNP region = Southern Nations, Nationalities, and Peoples’ region
